# Overweight, obesity and risk of liver cancer: a meta-analysis of cohort studies

**DOI:** 10.1038/sj.bjc.6603932

**Published:** 2007-08-14

**Authors:** S C Larsson, A Wolk

**Affiliations:** 1 Division of Nutritional Epidemiology, The National Institute of Environmental Medicine, Karolinska Institutet, PO Box 210, Stockholm SE-17177, Sweden

**Keywords:** body mass index, cohort studies, liver cancer, meta-analysis, obesity, review

## Abstract

Cohort studies of excess body weight and risk of liver cancer were identified for a meta-analysis by searching MEDLINE and EMBASE databases from 1966 to June 2007 and the reference lists of retrieved articles. Results from individual studies were combined using a random-effects model. We identified 11 cohort studies, of which seven on overweight (with a total of 5037 cases) and 10 on obesity (with 6042 cases) were suitable for meta-analysis. Compared with persons of normal weight, the summary relative risks of liver cancer were 1.17 (95% confidence interval (CI): 1.02–1.34) for those who were overweight and 1.89 (95% CI: 1.51–2.36) for those who were obese. This meta-analysis finds that excess body weight is associated with an increased risk of liver cancer.

Although relatively rare in the United States and other developed countries, liver cancer is the third most common cause of death from cancer worldwide (Parkin *et al*, 2005). It is rarely detected early and is often fatal within a few months of diagnosis. The 5-year survival rate is only about 6–11% (Coleman *et al*, 2003; Ries *et al*, 2006). The age-adjusted incidence and mortality rates of liver cancer have been increasing rapidly in the United States since the mid-1980s (Ries *et al*, 2006). While approximately half of this increase can be attributable to hepatitis C virus infection, a minimal or no increase has been related to hepatitis B virus and alcoholic liver disease (El-Serag and Mason, 2000; Hassan *et al*, 2002). Given that about half of the increase in liver cancer incidence is not related to hepatitis, the major risk factor in a significant proportion of the cases has yet to be identified.

Coinciding with the rising incidence of liver cancer, the prevalence of obesity has been increasing markedly over the past two decades worldwide (Larsson and Wolk, 2006). Obesity has been recognised as a risk factor for several malignancies, including cancer of the breast (in premenopausal women), endometrium, kidney (renal cell), colon, pancreas, gallbladder, and esophagus (adenocarcinoma) (IARC, 2002; Larsson *et al*, 2007; Larsson and Wolk, 2007). Accumulating epidemiologic evidence also indicates that excess body weight may be a risk factor for liver cancer, but the evidence has not been quantitatively summarised. We have therefore quantitatively assessed the associations of overweight and obesity with liver cancer risk in a meta-analysis of cohort studies.

## MATERIALS AND METHODS

### Study selection

A literature search was conducted in the MEDLINE and EMBASE databases for pertinent studies published in any language from 1966 to June 2007. We used the keywords ‘body mass index’, ‘BMI’, or ‘obesity’ in combination with ‘hepatocellular carcinoma’, ‘liver cancer’, or ‘liver neoplasm’. Moreover, we manually reviewed the reference lists of retrieved articles to search for more studies.

Studies were included in the meta-analysis if they fulfilled the following criteria: (1) cohort study in which liver cancer incidence or mortality was an outcome; (2) the exposure of interest was overweight and/or obesity defined by body mass index (BMI) (the weight in kilograms divided by the square of height in meters); and (3) relative risk estimates (rate ratio or standardized incidence ratio) with corresponding 95% confidence intervals (CIs) were reported.

### Data extraction

For each study, the following information was extracted: first author's last name; publication year; country in which the study was performed; sample size; method of assessing weight and height; type of outcome (incidence or mortality); variables adjusted for in the analysis; and the relative risks with 95% CIs for overweight and obesity *vs* normal weight. From each study, we extracted the most fully adjusted relative risks.

### Statistical analysis

The relative risks and corresponding standard errors (derived from the CIs) from individual studies were logarithmically transformed to stabilize variances and normalize the distributions. We calculated summary relative risks for overweight (defined as BMI 25–30 kg m^−2^) and obesity (BMI ⩾30 kg m^−2^) *vs* normal weight (BMI 18.5–24.9 kg m^−2^). For two studies (Calle *et al*, 2003; Kuriyama *et al*, 2005) that reported relative risks for two categories of BMI that fell into the category representing overweight or obesity, we pooled the relative risks and used the pooled estimate in the meta-analysis. Study-specific relative risks were combined using the DerSimonian and Laird random-effects model (DerSimonian and Laird, 1986). Thus, each summary relative risk was a weighted average of the study-specific relative risk, where the weight for each study is the inverse of the sum of the within-study variance for that study, and the between-study variance.

Statistical heterogeneity among studies was evaluated with the *Q* and *I*^2^ statistics (Higgins and Thompson, 2002). For the *Q* statistic, statistical significance was set at *P*<0.1. We used funnel plots (i.e. plots of study results against precision) to assess publication bias, and tested the symmetry of the funnel plot using Egger's test (Egger *et al*, 1997).

Results are presented graphically, whereby squares represent study-specific relative risks and diamonds represent summary relative risks. The area of each square is proportional to the inverse of the variance of the logarithm of the relative risk; 95% CIs for individual studies are represented by horizontal lines and for the summary estimates by the width of the diamonds. Statistical analyses were performed with Stata, version 9.0 (StataCorp, College Station, TX, USA).

Population attributable risk (PAR) for liver cancer was estimated for individuals with excess body weight (BMI⩾25 kg m^−2^) compared to those of normal weight (BMI<25 kg m^−2^). The PAR describes the theoretic proportion of cases that would be prevented if all individuals were moved into the exposure level associated with the lowest risk for that factor. The PAR (PAR%) was calculated as: PAR%=(*p* × [RR–1]/[*p* × (RR–1)+1]) × 100%, where *p* represents the prevalence in the population and RR the relative risk. Prevalence data were obtained from the National Health and Nutrition Examination Survey that assessed the prevalence of overweight and obesity in a representative sample of the US population (Ogden *et al*, 2006). In that survey, 39.7% of the men were overweight and 31.1% were obese. Among women, 28.6% were overweight and 33.2% were obese. PARs were calculated for the overweight and obese categories using the obtained summary relative risks, and then summarized across the two categories for men and women separately.

## RESULTS

We identified 11 eligible cohort studies (Møller *et al*, 1994; Wolk *et al*, 2001; Nair *et al*, 2002; Calle *et al*, 2003; Samanic *et al*, 2004, 2006; Batty *et al*, 2005; Kuriyama *et al*, 2005; Oh *et al*, 2005; Rapp *et al*, 2005; N'Kontchou *et al*, 2006), of which 7 on overweight (with a total of 5037 cases) (Calle *et al*, 2003; Batty *et al*, 2005; Kuriyama *et al*, 2005; Oh *et al*, 2005; Rapp *et al*, 2005; N'Kontchou *et al*, 2006; Samanic *et al*, 2006) and 10 on obesity (with a total of 6042 cases) (Møller *et al*, 1994; Wolk *et al*, 2001; Nair *et al*, 2002; Calle *et al*, 2003; Samanic *et al*, 2004, 2006; Batty *et al*, 2005; Oh *et al*, 2005; Rapp *et al*, 2005; N'Kontchou *et al*, 2006) were suitable for meta-analysis. Characteristics of the studies are shown in Table 1. Seven studies were conducted in Europe, two in the United States, and two in Asia. Weight and height were measured in six studies and self-reported in two studies; in three studies, obesity was defined by a discharge diagnosis of obesity. The outcome was incidence of liver cancer in all but two studies (Calle *et al*, 2003; Batty *et al*, 2005) in which the outcome was mortality from liver cancer. Two studies were based on standardized incidence ratio (Møller *et al*, 1994; Wolk *et al*, 2001). Two studies consisted of patients with cirrhosis (Nair *et al*, 2002; N'Kontchou *et al*, 2006).

**Figure 1:**
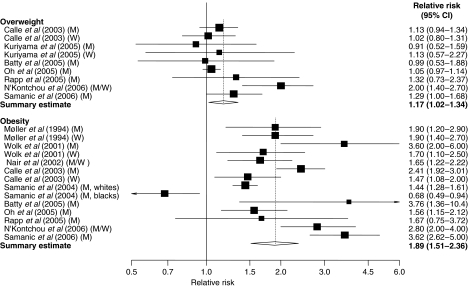
Relative risks of liver cancer associated with overweight and obesity. Relative risk estimates are for overweight and obese persons compared with normal weight persons. Tests for heterogeneity: overweight, *Q*=16.83, *P*=0.03; *I*^2^=52.5%; obesity, *Q*=88.03, *P*<0.001; *I*^2^=86.4%. M=men; W=women.

Relative risks of liver cancer for overweight and obese individuals compared to those of normal weight for individual studies (separately for men and women wherever this data were available) and all studies combined are shown in Figure 1. Meta-analysis of all studies found that compared to individuals with normal weight, those who were overweight or obese had a 17 and 89%, respectively, increased risk of liver cancer. There was statistically significant heterogeneity among the results of individual studies (Figure 1). The summary relative risk for obesity was statistically significantly higher (*P*=0.03) for men (RR: 2.42; 95% CI: 1.83–3.20; *n*=7 studies) than for women (RR: 1.67; 95% CI: 1.37–2.03; *n*=3 studies). There was no evidence for publication bias on the funnel plot (data not shown) or by Egger's test (*P*=0.31 for overweight and *P*=0.21 for obesity).

In a sensitivity analysis excluding the two studies that consisted of patients with cirrhosis (Nair *et al*, 2002; N'Kontchou *et al*, 2006), the summary relative risks were 1.07 (95% CI: 1.01–1.15) for overweight and 1.85 (95% CI: 1.44–2.37) for obesity. With stratification by assessment of obesity, the summary relative risks for the association between obesity and liver cancer were 2.15 (95% CI: 1.66–2.77) for studies based on measured or self-reported weight and height (Nair *et al*, 2002; Calle *et al*, 2003; Batty *et al*, 2005; Oh *et al*, 2005; Rapp *et al*, 2005; N'Kontchou *et al*, 2006; Samanic *et al*, 2006) and 1.61 (95% CI: 1.14–2.27) for studies based on a discharge diagnosis of obesity (Møller *et al*, 1994; Wolk *et al*, 2001; Samanic *et al*, 2004).

The PAR for excess body weight was calculated using the estimates of prevalence in the United States (Ogden *et al*, 2006) and the obtained summary relative risks of 1.17 and 1.89 for overweight and obesity, respectively. We estimated that 28% of liver cancer cases among men and 27% among women could be attributable to excess body weight (BMI⩾25 kg m^−2^).

## DISCUSSION

This is the first meta-analysis on overweight and obesity in relation to liver cancer risk and it indicates that excess body weight is associated with increased risk. Summary results showed that the risk was 17 and 89% higher among persons who were overweight and obese, respectively, compared with those of normal weight. The relation between obesity and liver cancer seemed to be stronger in men than in women.

Although there was statistically significant heterogeneity among study results, the relation between obesity and risk of liver cancer was consistent. Differences in the relative risk estimates were largely in the magnitude rather than the direction of the association. All but 1 out of the 14 relative risk estimates for the association between obesity and liver cancer were above one (ranging from 1.44 to 3.76), and 12 of these estimates were statistically significant.

A potential limitation of this meta-analysis is that individual studies may have failed to control for potential known or unknown confounders. The most important risk factors for the development of liver cancer are chronic infections with hepatitis B virus and hepatitis C virus. Heavy, long-term alcohol consumption is also a risk factor (Yu and Yuan, 2004). None of the studies adjusted for hepatitis B or C virus infections, and only three (Calle *et al*, 2003; Kuriyama *et al*, 2005; Oh *et al*, 2005) controlled for alcohol intake. It is unlikely, however, that these risk factors are strongly related to body weight and entirely explain the observed relationship between excess body weight and liver cancer risk. Another limitation is that we could not examine whether the association between excess body weight and liver cancer was modified by hepatitis virus infections and alcohol intake because the studies included in this meta-analysis did not provide results stratified by these factors.

As this meta-analysis was based on published studies, possible publication bias could have affected the results. However, neither funnel plots nor formal statistical tests showed evidence for publication bias.

The observed increased risk of liver cancer associated with excess body weight may be mediated through the development of non-alcoholic fatty liver disease (NAFLD), a chronic liver disease that occurs in non-drinkers. NAFLD is characterized by a spectrum of liver tissue changes, ranging from accumulation of fat in the liver to non-alcoholic steatohepatitis (NASH), cirrhosis, and liver cancer at the most extreme end of the spectrum. Up to 90% of obese individuals have some degree of fatty liver, and approximately 25–30% have NASH (Neuschwander-Tetri and Caldwell, 2003).

In summary, this meta-analysis supports evidence of an increased risk of liver cancer among overweight and obese persons. These findings indicate that liver cancer may, in part, be prevented by maintaining a healthy body weight.

## Figures and Tables

**Table 1:** Characteristics of cohort studies included in the meta-analysis

**Study**	**Country**	**No. of cases (men/women)**	**Study participants**	**Assessment of exposure**	**Adjustments**
Møller *et al* (1994)	Denmark	22/36	Men: 14 531 Women: 29 434	Discharge diagnosis of obesity	Age
Wolk *et al* (2001)	Sweden	15/13	Men: 8165 Women: 19 964	Discharge diagnosis of obesity	Age, calendar year
Nair *et al* (2002)	USA	659^a^	Men and women: 19 271^a^	Measured	Age, sex, race, diabetes
Calle *et al* (2003)	USA	620/345	Men: 404 576 Women: 495 477	Self-reported	Age, race, education, marital status, smoking, physical activity, aspirin use, estrogen-replacement therapy (women), alcohol, dietary factors
Samanic *et al* (2004)	USA	322 whites/38 blacks	White men: 3 668 486 Black men: 832 214	Discharge diagnosis of obesity	Age, calendar year
Kuriyama *et al* (2005)	Japan	69/31	Men: 12 485 Women: 15 054	Self-reported	Age, type of health insurance, smoking, intakes of alcohol, meat, fish, fruits, vegetables, bean-paste soup^b^
Batty *et al* (2005)	UK	51	Men: 18 403	Measured	Age, employment grade, marital status, physical activity, smoking, other^c^
Oh *et al* (2005)	Korea	3347	Men: 781 283	Measured	Age, area of residence, family history of cancer, smoking, exercise, alcohol
Rapp *et al* (2005)	Austria	57	Men: 67 447	Measured	Age, occupational group, smoking
N'Kontchou *et al* (2006)	France	220^a^	Men and women: 771^a^	Measured	Age, sex, cirrhosis cause, diabetes
Samanic *et al* (2006)	Sweden	297	Men: 362 552	Measured	Age, smoking

a Patients with cirrhosis.

b Odds ratios for women were further adjusted for age at menarche, age at end of first pregnancy, and menopausal status.

c Other factors adjusted for include disease at entry, weight loss in the last year, height-adjusted FEV_1_, triceps skinfold thickness, blood pressure-lowering medication, blood pressure, plasma cholesterol, glucose intolerance, and diabetes.

## References

[bib1] Batty GD, Shipley MJ, Jarrett RJ, Breeze E, Marmot MG, Smith GD (2005) Obesity and overweight in relation to organ-specific cancer mortality in London (UK): findings from the original Whitehall study. Int J Obes (Lond) 29: 1267–1274 15997248 10.1038/sj.ijo.0803020

[bib2] Calle EE, Rodriguez C, Walker-Thurmond K, Thun MJ (2003) Overweight, obesity, and mortality from cancer in a prospectively studied cohort of US adults. N Engl J Med 348: 1625–1638 12711737 10.1056/NEJMoa021423

[bib3] Coleman MP, Gatta G, Verdecchia A, Esteve J, Sant M, Storm H, Allemani C, Ciccolallo L, Santaquilani M, Berrino F (2003) EUROCARE-3 summary: cancer survival in Europe at the end of the 20th century. Ann Oncol 14(Suppl 5): v128–v149 14684503 10.1093/annonc/mdg756

[bib4] DerSimonian R, Laird N (1986) Meta-analysis in clinical trials. Control Clin Trials 7: 177–188 3802833 10.1016/0197-2456(86)90046-2

[bib5] Egger M, Davey Smith G, Schneider M, Minder C (1997) Bias in meta-analysis detected by a simple, graphical test. BMJ 315: 629–634 9310563 10.1136/bmj.315.7109.629 PMC2127453

[bib6] El-Serag HB, Mason AC (2000) Risk factors for the rising rates of primary liver cancer in the United States. Arch Intern Med 160: 3227–3230 11088082 10.1001/archinte.160.21.3227

[bib7] Hassan MM, Frome A, Patt YZ, El-Serag HB (2002) Rising prevalence of hepatitis C virus infection among patients recently diagnosed with hepatocellular carcinoma in the United States. J Clin Gastroenterol 35: 266–269 12192205 10.1097/00004836-200209000-00013

[bib8] Higgins JP, Thompson SG (2002) Quantifying heterogeneity in a meta-analysis. Stat Med 21: 1539–1558 12111919 10.1002/sim.1186

[bib9] IARC (2002) IARC handbooks of cancer prevention. Weight control and physical activity. Vol. 6, Lyon, France: IARC Press

[bib10] Kuriyama S, Tsubono Y, Hozawa A, Shimazu T, Suzuki Y, Koizumi Y, Ohmori K, Nishino Y, Tsuji I (2005) Obesity and risk of cancer in Japan. Int J Cancer 113: 148–157 15386435 10.1002/ijc.20529

[bib11] Larsson SC, Orsini N, Wolk A (2007) Body mass index and pancreatic cancer risk: A meta-analysis of prospective studies. Int J Cancer 120: 1993–1998 17266034 10.1002/ijc.22535

[bib12] Larsson SC, Wolk A (2006) Epidemiology of obesity and diabetes: prevalence and trends. In Obesity and Diabetes, Mantzoros C (ed) pp 15–36. Boston: Humana Press

[bib13] Larsson SC, Wolk A (2007) Obesity and the risk of gallbladder cancer: a meta-analysis. Br J Cancer 96: 1457–1461 17375043 10.1038/sj.bjc.6603703 PMC2360167

[bib14] Møller H, Mellemgaard A, Lindvig K, Olsen JH (1994) Obesity and cancer risk: a Danish record-linkage study. Eur J Cancer 30A: 344–350 8204357 10.1016/0959-8049(94)90254-2

[bib15] Nair S, Mason A, Eason J, Loss G, Perrillo RP (2002) Is obesity an independent risk factor for hepatocellular carcinoma in cirrhosis? Hepatology 36: 150–155 12085359 10.1053/jhep.2002.33713

[bib16] Neuschwander-Tetri BA, Caldwell SH (2003) Nonalcoholic steatohepatitis: summary of an AASLD Single Topic Conference. Hepatology 37: 1202–1219 12717402 10.1053/jhep.2003.50193

[bib17] N'Kontchou G, Paries J, Htar MT, Ganne-Carrie N, Costentin L, Grando-Lemaire V, Trinchet JC, Beaugrand M (2006) Risk factors for hepatocellular carcinoma in patients with alcoholic or viral C cirrhosis. Clin Gastroenterol Hepatol 4: 1062–1068 16844421 10.1016/j.cgh.2006.05.013

[bib18] Ogden CL, Carroll MD, Curtin LR, McDowell MA, Tabak CJ, Flegal KM (2006) Prevalence of overweight and obesity in the United States, 1999–2004. JAMA 295: 1549–1555 16595758 10.1001/jama.295.13.1549

[bib19] Oh SW, Yoon YS, Shin SA (2005) Effects of excess weight on cancer incidences depending on cancer sites and histologic findings among men: Korea national health insurance corporation study. J Clin Oncol 23: 4742–4754 16034050 10.1200/JCO.2005.11.726

[bib20] Parkin DM, Bray F, Ferlay J, Pisani P (2005) Global cancer statistics, 2002. CA Cancer J Clin 55: 74–108 15761078 10.3322/canjclin.55.2.74

[bib21] Rapp K, Schroeder J, Klenk J, Stoehr S, Ulmer H, Concin H, Diem G, Oberaigner W, Weiland SK (2005) Obesity and incidence of cancer: a large cohort study of over 145 000 adults in Austria. Br J Cancer 93: 1062–1067 16234822 10.1038/sj.bjc.6602819 PMC2361672

[bib22] Ries LAG, Harkins D, Krapcho M, Mariotto A, Miller AB, Feuer EJ, Clegg L, Eisner MP, Horner MJ, Howlader N, Hayat M, Hankey BF, Edwards BK (2006) SEER cancer statistics review, 1975–2003. Bethesda, MD: National Cancer Institute, 2006 http://seer.cancer.gov/csr/1975_2003/ (accessed 30 June 2007)

[bib23] Samanic C, Chow WH, Gridley G, Jarvholm B, Fraumeni JF (2006) Relation of body mass index to cancer risk in 362 552 Swedish men. Cancer Causes Control 17: 901–909 16841257 10.1007/s10552-006-0023-9

[bib24] Samanic C, Gridley G, Chow WH, Lubin J, Hoover RN, Fraumeni Jr JF (2004) Obesity and cancer risk among white and black United States veterans. Cancer Causes Control 15: 35–43 14970733 10.1023/B:CACO.0000016573.79453.ba

[bib25] Wolk A, Gridley G, Svensson M, Nyren O, McLaughlin JK, Fraumeni JF, Adam HO (2001) A prospective study of obesity and cancer risk (Sweden). Cancer Causes Control 12: 13–21 11227921 10.1023/a:1008995217664

[bib26] Yu MC, Yuan JM (2004) Environmental factors and risk for hepatocellular carcinoma. Gastroenterology 127: S72–S78 15508106 10.1016/j.gastro.2004.09.018

